# Supplementary material for: Machine Learning Accelerates Crystallization for Structure Determination
Source: Angew Chem Int Ed Engl. 2026 May 4;65(25):e1218503. doi: 10.1002/anie.1218503 (PMC13266958; doi:10.1002/anie.1218503)

checkCIF/PLATON report

Structure factors have been supplied for datablock(s) lcz107

THIS REPORT IS FOR GUIDANCE ONLY. IF USED AS PART OF A REVIEW PROCEDURE FOR PUBLICATION, IT SHOULD NOT REPLACE THE EXPERTISE OF AN EXPERIENCED CRYSTALLOGRAPHIC REFEREE.

No syntax errors found. CIF dictionary Interpreting this report

Datablock: lcz107

Bond precision:	C-C = 0.0053 A		Wavelength=1.54184
Cell:	a=12.91607(6)	b=14.44842(7)	c=22.98595(11)
	alpha=90	beta=91.3724(4)	gamma=90
Temperature:	100 K		

	Calculated	Reported
Volume	4288.33(4)	4288.34(3)
Space group	P 21/n	P 1 21/n 1
Hall group	-P 2yn	-P 2yn
Moiety formula	C34 H33 Ag3 F18 N6 O5	C15 H3 Ag3 F18 N6, C19 H30 O5
Sum formula	C34 H33 Ag3 F18 N6 O5	C34 H33 Ag3 F18 N6 O5
Mr	1271.28	1271.27
Dx,g cm-3	1.969	1.969
Z	4	4
Mu (mm-1)	12.051	12.051
F000	2488.0	2488.0
F000'	2499.34	
h,k,lmax	16,18,29	16,18,29
Nref	9236	8870
Tmin,Tmax	0.105,0.300	0.507,1.000
Tmin'	0.014	

Correction method= # Reported T Limits: Tmin=0.507 Tmax=1.000
AbsCorr = MULTI-SCAN

Data completeness= 0.960   Theta(max)= 78.461

R(reflections)= 0.0411(8785)   wR2(reflections)= 0.1128(8870)

S = 1.062   Npar= 600

The following ALERTS were generated. Each ALERT has the format
test-name_ALERT_alert-type_alert-level.
Click on the hyperlinks for more details of the test.

Alert level C

```
PLAT042_ALERT_1_C Calc. and Reported MoietyFormula Strings  Differ     Please Check
              Calc: C34 H33 Ag3 F18 N6 O5
              Rep.: C15 H3 Ag3 F18 N6, C19 H30 O5
PLAT213_ALERT_2_C Atom F5              has ADP max/min Ratio .....        3.4 prolat
PLAT213_ALERT_2_C Atom F00Q            has ADP max/min Ratio .....        3.4 prolat
PLAT213_ALERT_2_C Atom F00R            has ADP max/min Ratio .....        3.2 prolat
PLAT213_ALERT_2_C Atom F00S            has ADP max/min Ratio .....        3.4 prolat
PLAT213_ALERT_2_C Atom F014            has ADP max/min Ratio .....        3.9 prolat
PLAT213_ALERT_2_C Atom F017            has ADP max/min Ratio .....        3.1 prolat
PLAT213_ALERT_2_C Atom O00H            has ADP max/min Ratio .....        3.1 oblate
PLAT220_ALERT_2_C NonSolvent   Resd 1  F   Ueq(max)/Ueq(min) Range        3.6 Ratio
PLAT230_ALERT_2_C Hirshfeld Test Diff for    F6        --C01F     .        7.0 s.u.
PLAT230_ALERT_2_C Hirshfeld Test Diff for    F00V      --C01A     .        6.3 s.u.
PLAT242_ALERT_2_C Low    'MainMol' Ueq as Compared to Neighbors of       C01A Check
PLAT242_ALERT_2_C Low    'MainMol' Ueq as Compared to Neighbors of       C01F Check
PLAT242_ALERT_2_C Low    'MainMol' Ueq as Compared to Neighbors of       C01K Check
PLAT242_ALERT_2_C Low    'MainMol' Ueq as Compared to Neighbors of       C01R Check
PLAT911_ALERT_3_C Missing FCF Refl Between Thmin & STh/L=    0.600         14 Report
                0  2  0,  -1  0  1,   5 14  9,  12  0 16,  12  1 16,  -9 10 16,
                4 13 16,  -5  0 17,  11  0 17,   3 13 17,  10  1 18,  11  1 18,
               -8  9 19,  10  0 20,
PLAT971_ALERT_2_C Check Calcd Resid. Dens.  0.79Ang From F00V           2.00 eA-3
PLAT971_ALERT_2_C Check Calcd Resid. Dens.  0.62Ang From F00V           1.67 eA-3
PLAT972_ALERT_2_C Check Calcd Resid. Dens.  0.71Ang From Ag03          -2.00 eA-3
PLAT972_ALERT_2_C Check Calcd Resid. Dens.  0.79Ang From Ag01          -1.93 eA-3
PLAT972_ALERT_2_C Check Calcd Resid. Dens.  0.85Ang From Ag01          -1.87 eA-3
PLAT972_ALERT_2_C Check Calcd Resid. Dens.  0.67Ang From Ag02          -1.80 eA-3
PLAT972_ALERT_2_C Check Calcd Resid. Dens.  0.31Ang From F00V          -1.73 eA-3
PLAT972_ALERT_2_C Check Calcd Resid. Dens.  0.58Ang From Ag03          -1.71 eA-3
PLAT974_ALERT_2_C Check Calcd Negative Resid. Density on      Ag02      -1.23 eA-3
PLAT974_ALERT_2_C Check Calcd Negative Resid. Density on      Ag01      -1.15 eA-3
```

Alert level G

```
PLAT003_ALERT_2_G Number of Uiso or U(i,j)  Restrained non-H-Atoms           2 Report
PLAT004_ALERT_5_G Polymeric Structure Found with Maximum Dimension           1 Info
PLAT007_ALERT_5_G Number of Unrefined Donor-H Atoms .............           2 Report
              H00B  H00L
PLAT083_ALERT_2_G SHELXL Second Parameter in WGHT  Unusually Large      15.29 Why ?
PLAT142_ALERT_4_G s.u. on b - Axis Small or Missing ..............    0.00007 Ang.
PLAT143_ALERT_4_G s.u. on c - Axis Small or Missing ..............    0.00011 Ang.
PLAT145_ALERT_4_G s.u. on beta     Small or Missing ..............     0.0004 Degree
```

```
PLAT186_ALERT_4_G The CIF-Embedded .res File Contains ISOR Records          1 Report
PLAT720_ALERT_4_G Number of Unusual/Non-Standard Labels ..........         95 Note
              Ag01    Ag02    Ag03    F004    F005    F006    F007    F008
              O009    O00A    O00B    H00B    F00C    N00D    N00E    N00F
              N00G    O00H    N00I    F00J    O00L    H00L    N00N    F00O
              F00P    F00Q    F00R    F00S    C00T    C00U    H00U    F00V
              C00W    C00X    C00Y    H00Y    C00Z    C011    C012    C013
              H013    F014    C015    H015    C016    F017    C018    C019
              C01A    C01B    C01C    H01A    H01B    C01D    C01E    H01C
              H01D    C01F    C01G    C01H    C01I    H01E    H01F    C01J
              H01G    H01H    C01K    C01L    C01M    H01I    H01J    C01N
              H01K    H01L    C01O    H01M    H01N    H01O    C01P    H01P
              H01Q    C01Q    H01R    H01S    H01T    C01R    C01S    H01U
              H01V    H01W    C01T    H01X    H01Y    C01U    H01Z
PLAT860_ALERT_3_G Number of Least-Squares Restraints .............         12 Note
PLAT912_ALERT_4_G Missing # of FCF Reflections Above STh/L=  0.600        352 Note
PLAT941_ALERT_3_G Average HKL Measurement Multiplicity ...........        2.7 Low
PLAT969_ALERT_5_G The 'Henn et al.' R-Factor-gap value ...........      5.085 Note
              Predicted wR2: Based on SigI**2  2.22 or SHELX Weight 10.62
PLAT978_ALERT_2_G Number C-C Bonds with Positive Residual Density.          1 Info
```

```
   0 ALERT level A = Most likely a serious problem - resolve or explain
   0 ALERT level B = A potentially serious problem, consider carefully
  26 ALERT level C = Check. Ensure it is not caused by an omission or oversight
  14 ALERT level G = General information/check it is not something unexpected

   1 ALERT type 1 CIF construction/syntax error, inconsistent or missing data
  27 ALERT type 2 Indicator that the structure model may be wrong or deficient
   3 ALERT type 3 Indicator that the structure quality may be low
   6 ALERT type 4 Improvement, methodology, query or suggestion
   3 ALERT type 5 Informative message, check
```

Validation response form

Please find below a validation response form (VRF) that can be filled in and pasted into your CIF.

```
# start Validation Reply Form
_vrf_PLAT042_lcz107
;
PROBLEM: Calc. and Reported MoietyFormula Strings  Differ     Please Check
RESPONSE: ...
;
_vrf_PLAT213_lcz107
;
PROBLEM: Atom F5              has ADP max/min Ratio .....        3.4 prolat
RESPONSE: ...
;
_vrf_PLAT220_lcz107
;
PROBLEM: NonSolvent   Resd 1  F   Ueq(max)/Ueq(min) Range        3.6 Ratio
RESPONSE: ...
;
_vrf_PLAT230_lcz107
;
PROBLEM: Hirshfeld Test Diff for    F6       --C01F     .        7.0 s.u.
```

```
RESPONSE: ...
;
_vrf_PLAT242_lcz107
;
PROBLEM: Low    'MainMol' Ueq as Compared to Neighbors of       C01A Check
RESPONSE: ...
;
_vrf_PLAT911_lcz107
;
PROBLEM: Missing FCF Refl Between Thmin & STh/L=    0.600         14 Report
RESPONSE: ...
;
_vrf_PLAT971_lcz107
;
PROBLEM: Check Calcd Resid. Dens.  0.79Ang From F00V           2.00 eA-3
RESPONSE: ...
;
_vrf_PLAT972_lcz107
;
PROBLEM: Check Calcd Resid. Dens.  0.71Ang From Ag03          -2.00 eA-3
RESPONSE: ...
;
_vrf_PLAT974_lcz107
;
PROBLEM: Check Calcd Negative Resid. Density on      Ag02      -1.23 eA-3
RESPONSE: ...
;
# end Validation Reply Form
```

It is advisable to attempt to resolve as many as possible of the alerts in all categories. Often the minor alerts point to easily fixed oversights, errors and omissions in your CIF or refinement strategy, so attention to these fine details can be worthwhile. It is up to the individual to critically assess their own results and, if necessary, seek expert advice.

PLATON version of 17/09/2025; check.def file version of 17/09/2025

---

duplicate check

No duplication found

Datablock lcz107 - ellipsoid plot

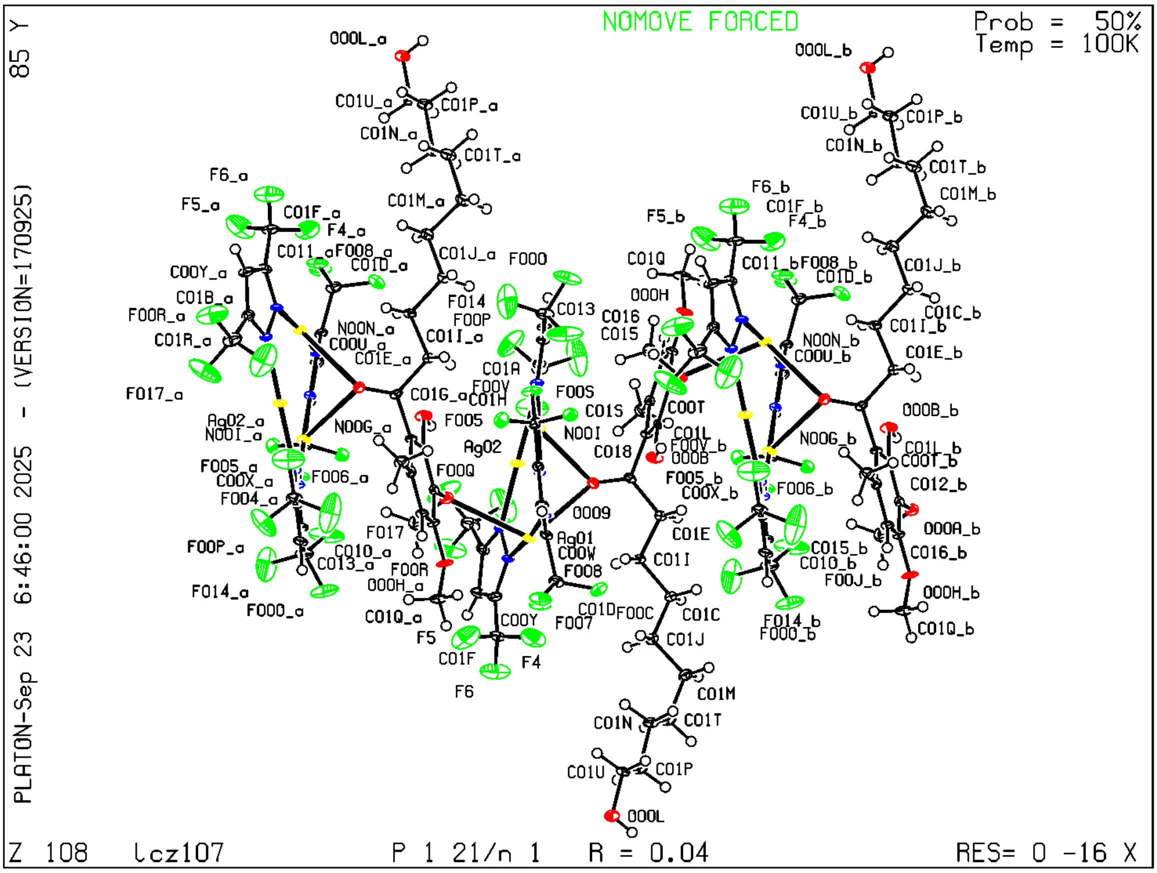

Supplement: Supplementary file 2 — Supporting File 2: anie72462‐sup‐0002‐DataFile.zip. [file ANIE-65-e1218503-s002.zip › supplementary_materials_crystallographic_data submit.zip!/cif_and_checkcif(1-114)/Ag3Pz3_107/Ag3Pz3_107_checkcif.pdf]
